# The Impact of Oxygen at Various Stages of Vinification on the Chemical Composition and the Antioxidant and Sensory Properties of White and Red Wines

**DOI:** 10.1155/2020/7902974

**Published:** 2020-03-27

**Authors:** Tomasz Tarko, Aleksandra Duda-Chodak, Paweł Sroka, Małgorzata Siuta

**Affiliations:** Department of Fermentation Technology and Microbiology, University of Agriculture in Krakow, Faculty of Food Technology, ul. Balicka 122, 30-149 Krakow, Poland

## Abstract

The purpose of this review was to collect and systematize information on the role and importance of oxygen in winemaking. Both the positive and negative effects of oxygen are presented and discussed throughout the text. The review characterizes the subsequent stages of the wine production process, during which oxygen comes into contact with fruits, must, and finally wine. The impact of oxygen on the growth and metabolism of yeast, on the activity of enzymes, and on the final quality of wine was presented. The discussion of the effect of oxygen presence on the taste, aroma, colour, and stability contains a detailed description of changes of volatile compounds, polyphenols, and other important components of wine that take place in the presence of oxygen in both white and red wines. New techniques based on the use of oxygen to obtain the desired sensory characteristics of wine were also presented.

## 1. Introduction

Wine making depends on many factors related to the cultivation of vines and the process of its production. Location and therefore climatic conditions (insolation, precipitation, and temperature) and the way the vineyard operates affect the chemical composition of grapes, and the appropriate selection of the variety to the climatic conditions of a given region results in obtaining a good raw material for wine production. The process of vinification is fundamental for wine quality. Although the fermentation is an anaerobic process, oxygen is present at various stages of the winemaking process, and more attention has been paid to its impact and its pivotal role has been emphasized more often. Individual stages of the production process differ in terms of oxygen exposure, with sedimentation and fermentation as the least oxygen processes.

The aim of this paper is to show the importance of oxygen in the vinification process and to systematize information on the influence of oxygen on wine quality during the individual stages of its production.

## 2. Initial Stages of Vinification

Oxygen is an important factor in creating quality wines as it takes part in enzymatic and nonenzymatic oxidation reactions. The intensity of these transformations depends on many factors, including the concentration and structure of phenolic compounds which are the substrates of the reaction [1]. Red wines contain higher concentrations of phenolic compounds; hence, they are easier to oxidize, but their amount (despite oxidation) makes wines more resistant to oxygen. Therefore, the contact with oxygen in the case of red wines is not as undesirable as in the case of white wines. White wines are very sensitive to oxidation due to small amounts of phenolic compounds [2]. Due to these differences, the impact of oxygen on wine is assessed differently depending on the type of wine. In the case of red wine production, some amount of oxygen absorbed during its production is desirable, contributing to the formation of desired sensory characteristics. Oxygen in the production of white wines is generally perceived as an undesirable factor. It causes adverse changes in the aroma, colour, and taste of the wine [3].

Both enzymatic and nonenzymatic oxidation of must in the case of the production of white wine is not recommended due to adverse changes in taste, colour, and aroma (Table 1). These changes result from the degradation of phenolic compounds, precursors of flavours, aromas, and colours. The progressive polymerization of phenolic compounds in must causes the formation of yellow to brown pigments. These reactions, therefore, change the colour of young white wine from slightly yellow to dark yellow, and even to an unacceptable brown. Even small amounts of oxygen at the stage of obtaining must for white wine can also lead to the loss of various aromas, especially those corresponding to the fruity aroma of white wines [7, 8].

In the case of red wines, enzymatic oxidation is also undesirable and may develop deposits, resulting in a reduction in the amount of phenolic compounds in the wine [6].

Phenolic compounds in intact fruits are mostly found in vacuoles, but as a result of their damage (at the stage of harvesting and subsequent stages of fruit processing), these compounds are released. Consequently, enzymes belonging to the oxidoreductase class may cause oxidation of these compounds (in particular hydroxybenzoic and hydroxycinnamic acid esters, such as coumaric acid and caftaric acid) [[3]; Morales et al., 2015].

Among the enzymes responsible for the enzymatic oxidation of phenolic compounds in juice, the most important are polyphenol oxidases, such as laccase, and peroxidase. Polyphenol oxidases, naturally occurring in grapevine fruit, have various activities: o-phenol hydroxylase causes monophenol to convert into monocatechol and catecholase participates in catechol oxidation to brown melanin, while laccase catalyzes the oxidation of p-hydroquinones to p-benzoquinones, with the use of hydrogen peroxide as an electron acceptor, resulting in an oxidized donor and water [6, 9]. Peroxidase is an Fe-containing enzyme whose activity depends on the available hydrogen peroxide (H_2_O_2_). However, the browning caused by peroxidase seems insignificant in fruits (with few exceptions), although some researchers found that it did enhance the degradation of phenols when coexisting with polyphenol oxidase [9].

The mechanism of enzymatic oxidation is based on the oxidation of phenolic compounds by polyphenol oxidase to caffeoyltartaric acid o-quinones (CTAQ). o-Quinones may undergo further reactions depending on their redox potential and electron affinity [6]. They can react with glutathione (GSH), naturally occurring in grapes, resulting in the formation of 2-S-glutathionyl caftaric acid (GRP). GRP limits to a certain extent the reaction of the formation of brown pigments, because o-quinones associated with glutathione as GRP are no longer a substrate that polyphenol oxidase can oxidize [10]. On the other hand, GRP is a substrate that laccase can oxidize by adding a glutathione molecule to GRP to form 2,5-S-diglutathionylcacaftaric acid (GRP2). The depletion of GRP, which prevents wine colour from changing by the reaction with o-quinones and other compounds of a nucleophilic nature, causes that the remaining o-quinones to oxidize other compounds of must, being reduced to the original phenolic compound, which again can be oxidized by polyphenol oxidase [11, 12].

The formed quinones can also polymerize and condense with many other compounds and lead to the formation of brown pigments, especially at the elevated pH [9]. o-Quinones are oxidants and can also cause oxidation of other substrates with lower redox potential, such as other phenolic compounds, ascorbic acid, and SO_2_, and during this process, quinones are reduced to phenols [6, 10].

Nonenzymatic oxidation reactions can be also observed in must which also results in the formation of o-quinones, but due to the slow rate of the process, enzymatic oxidation plays a more important role [2].

One of the methods to protect must against the harmful effects of oxygen is the use of SO_2_. It plays two roles: it prevents enzymatic and nonenzymatic oxidation and limits the growth of microorganisms. It inhibits the enzymatic oxidation reaction as a result of the slowdown of polyphenol oxidase or its almost complete inactivation. Sulphur dioxide addition in the amount of 50 mg/L causes a reduction in polyphenol oxidase activity in 75-90%. Sulphur dioxide may also limit chemical oxidation reactions by reducing the oxidation products to their original form (e.g., o-quinones to phenols). The effect on nonenzymatic oxidation reactions is based on the reaction with hydrogen peroxide, but not directly on the reaction with oxygen (in the typical wine pH, this reaction occurs very slowly and is not significant) [10, 13–16].

Another antioxidant used in viticulture is glutathione. It occurs naturally in the grapes and reduces enzymatic browning through reactions with the o-quinones formed by oxidation. The formed GRP is a colourless compound, and while reacting with o-quinones, it protects must against browning, as o-quinones could lead to the formation of brown pigments as a result of polymerization. Hence, glutathione has a protective role in relation to not only the colour but also the aroma of wines. In the case of white Sauvignon Blanc wines, glutathione by binding o-quinones prevents their binding to compounds such as 3-sulphanylhexan-1-ol, 3-sulphonylo hexyl acetate, and 4-sulphanyl-4-methylpentan-2-one responsible for the aroma of tropical fruit, due to which it prevents the loss of aroma [17, 18].

Ascorbic acid is naturally present in fruits and can be added during the process of winemaking, but it is usually rapidly consumed after crushing, typically due to either scavenging oxygen or reducing o-quinone derivatives. Ascorbic acid is highly reducing, with its reduction potential being estimated as ~210 mV (Ag/AgCl) at pH 3.6 by cyclic voltammetry, much lower than common polyphenols [9]. It limits oxidation by the direct or indirect reaction with oxygen which causes reduction of quinones to phenols [19]. The by-products of this process are dehydroascorbic acid and hydrogen peroxide, which is a strong oxidant and can lead to the oxidation of wine compounds [10, 20]. Further oxidation of dehydroascorbic acid causes its decomposition into numerous compounds, including 2-keto-L-xylose, which is a precursor of xanthene cations causing the colour change of wine [21]. It is recommended to use small doses (50-150 mg/L) of ascorbic acid to achieve the desired antioxidant properties, preferably in combination with SO_2_, which in the form of HSO^3-^ reacts with hydrogen peroxide [22, 23]. To limit the oxidation of must, inert gases are often used. Among them, carbon dioxide, nitrogen, and argon can be distinguished. They fill free spaces in pipes, tanks, etc. and therefore reduce the contact of must and wine with oxygen [1].

However, it should be noted that in some cases, the access of oxygen to must in a controlled manner can have positive effects. In the case of red wine production, oxygen in the maceration stage allows for an increased extraction of phenolic compounds from fruit tissues. The exposure to oxygen, although generally considered harmful to white wine, also has positive effects (Table 1). One of the methods to prevent the creation of an unfavourable brown colour of white wine is a technique called hyperoxidation. It involves the exposure of must to oxygen, resulting in rapid conversion of phenolic compounds, especially proanthocyanidins, contained in white must to brown polymerized compounds, as a result of the polyphenol oxidase activity. These compounds can then be easily removed during the clarification or fermentation stage. According to [10], the concentration of 9 mg O_2_/L is sufficient for flavonoids to be precipitated at a concentration of less than 100 mg/L. Since the oxidation of phenolic compounds in finished wine causes a colour change into brown, which is unacceptable for the consumer, their removal through the deliberate initiation of the enzymatic oxidation reaction at the prefermentation stage eliminates the substrates involved in the reactions of nonenzymatic oxidation. White wines obtained in this method are resistant to browning, even after bottling [4]. Hyperoxidation can be involved in the creation of desired sensory characteristics associated with the removal of flavonoids (such as catechin, epicatechin, and procyanidins B2 and B3) which, in white wine, could be responsible for the feeling of excessive acidity and astringency, as well as increased susceptibility to browning. In addition, flavonoid transformations in nonenzymatic oxidation reactions may lead to the loss of the characteristic aroma [7]. According to [7], hyperoxidation can cause both favourable and undesirable changes in aroma depending on the grape variety. For some types of wines (Chardonnay, Parrelada, and Muscat from Spain), some improvement in aroma was observed through the formation of higher concentrations of six-carbon compounds (hexane-1-ol, 2-hexenal), higher alcohols (2-phenylethanol), fatty acids, and their acetate and ethyl esters (hexyl, isoamyl, and 2-phenylethyl acetate and butyrate, decanoate, and ethyl hexanoate) as well as volatile terpenes favourably affecting the aroma of these wines. These compounds are responsible for the freshness of wines which is associated with the formation of fruity notes. In the case of Chardonnay, Mauzac, and Chenin from France, no changes in the aroma were observed, while wines produced from several European varieties resulted in a lower intensity of aroma due to the formation of acetates and higher aldehydes (containing from five to ten carbons) and lower concentrations of higher alcohols [1, 7]. On the other hand, other studies [18] have indicated that wines produced with the use of hyperoxygenation may lose their characteristic fruity aroma.

## 3. The Fermentation Stage

Oxygen is essential for the synthesis of lipids, the proper composition of which in the yeast cell membrane affects the maintenance of the cell membrane's integrity, obtaining a high glycolysis index and the highest possible yield of ethanol production (Table 2) [1, 24]. The lack of oxygen at the beginning of fermentation results in a decrease in fatty acid desaturase activity, as a result of which long-chain unsaturated fatty acids do not form (the oxygen molecule is necessary to form an unsaturated bond) [[24–26]]. The concentration of ergosterol also decreases, while the synthesis of squalene increases, which contributes to the inhibition of sterol biosynthesis [27]. In addition to squalene, lanosterol and zymosterol are also formed [26], as molecular oxygen is essential for converting squalene to ergosterol (squalene⟶lanosterol⟶zymosterol⟶ergosterol). The appropriate oxygen concentration and thus the chemical composition of cell membranes (high concentration of ergosterol and unsaturated fatty acids in the cell wall) determine the yeast cell's resistance to ethanol synthesized during fermentation [24].

In oxygen-free conditions, where lipid synthesis is impossible, the source of lipids is must [5]. Depending on the pressing and clarification techniques used, must may contain various amounts of phytosterols (*β*-sitosterol, campesterol, and stigmasterol) and fatty acids (palmitic, oleic, linoleic, and linolenic acids), which are essential for the growth and development of yeast [28]. These can be substitutes for ergosterol, and their use leads to an increase in yeast growth and fermentation activity. However, if no oxygen is added, these phytosterols can disrupt the proper membrane properties (these lipids replace the yeast-forming compounds and form the basis for membrane formation) and inhibit fermentation and its efficiency [5, 29]. In order to prevent these phenomena, the fermenting must be oxygenated [27] and the time point of oxygen addition is also important. The addition of oxygen before fermentation allowed obtaining a proper cellular structure of the yeast and resulted in a shorter fermentation time and better yeast viability in a high ethanol concentration environment [26]. The addition of oxygen during the first 48 hours of fermentation (along with the use of other nutrients) resulted also in a higher yield of ethanol production by yeast (17.89% without oxygenation and 20.96% with its application) [1, 30]. According to [31], the addition of oxygen in the amount of 10-20 mg/L in the final phase of yeast growth prevents fermentation from halting. In turn, [29] stated that in order to counteract halting fermentation due to improper membrane formation, oxygen should be added necessarily at the end of the growth phase in the amount of 5-10 mg/L.

Although the fermentation takes place under oxygen-free conditions (fermentation process: Embden-Meyerhof-Parnas pathway), the controlled addition of oxygen results in the desired sensory characteristics, such as colour and aroma of the wines, and also reduces the formation of reducing aromas. Oxygen affects the formation of volatile compounds, such as esters (acetates and ethyl esters), higher alcohols, medium-chain fatty acids, branched acids, aldehydes, and ketones [26]. Higher alcohols, also formed as a result of fermentation, are ester precursors. Their presence positively affects the complexity of wine aromas, but in concentrations above 300 mg/L, these can cause an intense, pungent aroma. During maturation, these can also be oxidized to aldehydes, which also affects wine aroma. Fatty acids, especially acetic, hexanoic, octanoic, and decanoic, contribute to the creation of a fresh aroma of wine. However, too high concentrations are unfavourable, and wines with a high proportion of them are perceived as rancid and cheese-like. These compounds are produced at an early stage of fermentation, especially when using highly clarified musts and in the case of total lack of oxygen [32, 33].

Oxygenation of must increases the concentration of esters such as acetates (propyl, isobutyl, isoamyl, 2-phenylethyl, and ethyl) and ethyl esters (ethyl propanoate, ethyl isobutanoate, ethyl butanoate, ethyl hexanoate, ethyl octanoate, ethyl decanoate, ethyl laurate, ethyl palmitate, ethyl pyruvate, and ethyl lactate) as well as butyl lactate and hexyl lactate. The amount of higher alcohols (propanol, isobutanol, isoamyl alcohols, phenylethyl alcohol, butanol, hexanol, heptanol, octanol, and decanol) also rises. An increase in the concentration of esters while maintaining a relatively low concentration of higher alcohols may lead to the intensification of the fruity aroma [34]. With regard to volatile compounds, [35] obtained different results. They showed that the content of volatile compounds significantly depends on the yeast strain, and under partial oxygenation of the must, lower concentrations of most esters and acids were obtained, while higher amounts of alcohols were observed [35].

Aldehydes are formed as a result of alcohol oxidation at the stage of fermentation and maturation of wines [1]. Aldehydes, mainly acetic aldehyde, and ketones in red wine are responsible for the formation of condensed compounds: tannins linked by an ethyl bridge and adducts of tannins and anthocyanins. Aldehydes also initiate many other reactions, including the transformation of malvidin 3-O-glucoside to pyranoanthocyanin—vitisin B, which is not discoloured due to the effect of SO_2_ and pH changes, thus resulting in colour stability. In addition, acetaldehyde and heterocyclic acetals are compounds that form the aroma of aged Madeiras and Ports [36, 37]. The aldehydes and esters have a low perceptibility threshold; therefore, even in small amounts, these can significantly worsen the aroma of wine. Particularly susceptible to the changes in the quality of aroma are wines produced from less aromatic fruits, which due to the low concentration of aroma-forming compounds are oxidized faster [38]. Aromas which are perceptible in oxidized white wine are described as the smell of caramel, rotten fruit, wood, and cooked vegetables [13, 39].

As a result of the oxidation of higher alcohols, aldehydes are formed, e.g., 3-(methylthio)propionaldehyde, which create the aroma of honey and boiled vegetables. In oxidized white wines, an increase in the concentration of 5-hydroxymethylfurfural and 5-methylfurfural and other furanaldehydes is observed. These compounds cause the formation of wood aroma, although white wines are not usually matured in barrels. 5-Methylfurfural and 5-hydroxymethylfurfural at high concentrations are characteristic of Port and Madeira wines, where these accumulate as a result of high temperature during aging in oak barrels [13, 40]. The oxidation of ethanol by hydrogen peroxide causes the formation of acetaldehyde, which constitutes the largest amount of the aldehydes formed. The aroma caused by acetaldehyde is associated with an acrid, grassy smell and adversely affects the aroma of wine [13, 41]. In turn, [1] state that even high concentrations of acetaldehyde do not affect the aroma of wine. As a result of oxidation, there is also an increase in the concentration of benzoic aldehyde (due to the oxidation of phenylalanine) and hexenal [1, 38]. The chemical and sensory impact of a microoxygenation treatment is highly dependent on the absence or presence of yeast growth. In microoxygenated Merlot wine, viable *Saccharomyces cerevisiae* yeasts caused a dramatic increase in acetaldehyde levels leading to significant sensory changes [42].

With the increase of oxygen concentration at the stage of yeast inoculation, the concentration of volatile thiols (4-mercapto-4-methylpentan-2-one and 3-mercaptohexan-1-ol) increases as yeasts are responsible for the release of volatile thiols from their nonvolatile, odourless precursors. The concentration of these compounds depends on the balance of alcohol acetyltransferase and esterase activity. These compounds cause the aroma of fruit, especially Sauvignon Blanc white wines, described in the literature as grapefruit aromas, guavas, and tropical fruits [5]. There are three mechanisms that cause these changes. In the presence of oxygen and iron ions, thiols are oxidized to the corresponding disulphides. As nucleophilic compounds, these can also be added to electrophilic compounds, such as polymerized phenolic compounds, and can also react with oxidation products of phenolic compounds, i.e., quinones. The resulting adducts are nonvolatile compounds and cause loss of aroma [13].

In addition, oxygen affects the malolactic fermentation that occurs after proper fermentation. Oxygenation of samples results in a faster conversion of malic acid into lactic acid, as well as in a change in sensory characteristics. Oxygen accelerates the conversion of *α*-acetoacetate to diacetyl, which contributes to the characteristic aroma of Chardonnay. However, little research has been done in this area; hence, these mechanisms are not fully understood [1].

## 4. Maturation of Wine

As a result of the oxidation reactions taking place in wine, there are changes in the aroma, colour, and taste that may have both positive and negative significance. It is important that the amount of oxygen coming into contact with the wine is appropriate and the transformations take place over a relatively long period of time [2]. It is thought that nonenzymatic oxidation contributes to a reduction in the quality of white wine, due to browning and adverse changes in aroma, but in the case of red wines, it allows for an intense and stable colour and improves the taste properties [43, 44]. Red wines undergo favourable transformations when oxygenated with more than 60 mg/L, while their quality decreases after the introduction of 150 mg O_2_/L [45].

Nonenzymatic oxidation of polyphenols is a process that occurs due to the presence of compounds with a catechol ring such as (+)-catechin, (-)-epicatechin, (+)-catechin gallate, gallic acid and its esters, caffeic acid, and anthocyanins [40]. Malvidin, the main coloured compound belonging to anthocyanins in red wines, p-coumaric acid, and resveratrol are oxidized at a higher redox potential [9, 46]. In chemical oxidation, oxygen does not react directly with phenolic compounds. Followed by the addition of a single electron transition of metal ions to the oxygen, which leads to the formation of superoxide anion O_2_^·−^, which at the pH of wine is present in the form of a hydroperoxyl radical HOO^·^, this radical causes the oxidation of phenolic compounds to quinones, and it can be reduced to hydrogen peroxide [9, 47]. Sulphur dioxide may react with hydrogen peroxide (resulting in sulphate (VI) and water) or with quinone causing its reduction to the original phenolic compound or forming a product with a sulphone group [47].

Hydrogen peroxide combined with iron ions generates a hydroxyl radical HO^·^ which is able to oxidize almost every organic molecule and many inorganic compounds, e.g., ethyl alcohol, tartaric acid, glycerol, sugars, and organic acids. The oxidation of ethanol leads to the formation of acetaldehyde, while tartaric acid oxidizes to dihydroxyfumaric acid, which forms yellow xanthan ions by reaction with (+)-catechin [9].

Quinones are formed as a result of oxidation of polyphenols and, due to high electrophilicity, may then spontaneously bind to certain phenols, thiols, and amines [9].

Wine may come into contact with small amounts of oxygen during aging in barrels or bottles and during filtration, centrifugation, and bottling (Table 3). According to [10], during the filtration process, the oxygen amount is 2-4 mg/L and, during centrifugation, 0.95 mg/L, while other sources [1] give the following values: 4-7 mg/L and above 8 mg/L, respectively. In the course of the maturation of wine in barrels, approx. 20-45 mg O_2_/L/year can penetrate through wood pores [1, 48], and its concentration may be 20-50 *μ*g/L [49].

Maturation of red wines usually lasts longer than 6 months and is traditionally carried out in barrels, which enables the transformation of phenolic compounds of wine as a result of the effect of oxygen penetrating the oak staves, as well as the extraction of wood compounds, including ellagotanins in amounts above 250 mg/L [48, 50, 51]. An alternative and much cheaper solution is microoxidation, consisting of controlled oxygenation of wines (2-9 mg O_2_/L/month), although, according to [52], a suitable dose of oxygen for the microoxidation of red wines is as much as 15 mL/L/month. This type of action allows induction of the desired changes in a much shorter time than during the classic maturation in barrels. They can be divided into a structural phase (tannins become more perceptible, wine flavour is perceived as more tart, and the intensity of the aroma is reduced) and the phase of harmonization (tannins are perceived as more gentle, the wine becomes more delicate, and the aroma is strengthened and gains more variety). A “tart taste” in wine is usually sharp or sour and leaves a taste in the mouth that can be tangy and tingling. The sensory characteristics of tannins depend on their structure—those with a higher molecular weight are perceived as tart, while those with a smaller molecular weight (dimers and trimers) increase acidity [41, 48, 53]. The feeling of astringency results from the interaction between the polymerized phenolic compounds and the saliva proteins [54], but only tannins with a molecular weight of 1000-3000 daltons are able to induce these reactions [Herderich & Smith, 2005]. Recent studies [55] have shown that astringency is primarily associated with the content of malic acid. Wines rich in malic acids showed the highest reactivity towards saliva proteins and a potential higher astringency. Excessive oxidation may lead to unfavourable changes that trigger transformations of compounds responsible for aroma, which result in a “flat” taste (wine that is lacking acidity, particularly on the finish), the loss of fruity notes, and may also lead to the occurrence of oxidation aroma [56]. These are associated with sulphur compounds and are caused by the formation of, e.g., hydrogen sulphide, methanethiol, ethanethiol, and thioacetates. In this case, the aroma of wine is described as the smell of mould, fungi, dirty cloth, rotten eggs, garlic, or cauliflower [5].

Acetaldehyde and quinones, formed through the oxidation of flavonoids and ethanol, accelerate the condensation of tannins with anthocyanins and flavan-3-oles [48, 56]. The high molecular compounds that are formed may fall out in the form of lees resulting in their decrease in concentration. The wine obtained is perceived as less tart. Lowering astringency is also caused by the fact that the formed tannin-anthocyanin adducts react with salivary proteins to a lesser extent [48].

The reactions of depolymerization can occur at low pH. As a consequence of breaking bonds between subunits of tannins, compounds of lower molecular weight are formed and released—hence, mature wine tastes less tart, whereas acidity is more sensible [54, 57]. In the case of red wines, astringency is perceived as a characteristic and positive if the sensation is balanced by the appropriate alcohol and extract content. A higher concentration of ethanol contributes to a more tart taste [48].

The amount of oxygen absorbed in the bottling stage is about 1-3 mg/L and depends on the method of bottling, primarily the use of inert gases, the type of machine used, and the capping system. The amount of free space left in the bottle is also important because 8 mL corresponds to a concentration of 3.2 mg O_2_/L wine [10]. The amount of oxygen absorbed during bottling and this found in the free space in a bottle (Total Oxygen Package (TPO)) usually reaches a value of 1-9 mg/L [13, 48].

Oxygen penetrates through the natural cork during the aging in bottles in an amount of 0.005-5 mg/L/year [48]. However, [58] proved that the rate of oxygen migration depended on the type of closure used and it was as follows: screw cap<synthetic coextruded<microagglomerated cork<natural cork. In studies of [10, 59], the highest oxygen permeation occurred through synthetic stoppers (up to 9.8 mg/L), then natural corks (5.9-8.3 mg/L), and agglomerated corks (below 3 mg/L) and the lowest in the case of caps. Small oxygen doses may cause favourable changes, further maturation of the wine, and decomposition of sulphur compounds responsible for the smell of burned rubber or reducing aromas, but too much or too little access of oxygen may have an adverse effect [57].

During wine maturation, there are many transformations within volatile compounds and the influence of oxygen at this stage is very important for the aroma of wine. The contact with oxygen causes an increase in the concentration of 1,1,6-trimethyl-1,2-dihydronaphthalene (TDN), which results in the aroma of gasoline in Riesling wines, as well as 2,6-dimethyl-7-octene-2,6-diol and 3-hydroxy-4,5-dimethyl-2(5H)-furanone (sotolon) [1]. Sotolon is a compound belonging to the group of lactones, which in high concentrations causes the aroma of curry, while in smaller—maple syrup, burnt sugar, and caramel. It is a characteristic and desirable compound, among others for Port and Sherry wines; however, it has an adverse effect for the aroma of dry white wines. It may be formed as a result of oxidation of ascorbic acid, which is the most probable mechanism for dry white wines [13].

Monoterpenes, especially geraniol, linalool, and alpha-terpineol, create floral, fruity, and citrus notes. The amount of these monoterpenes decreases as a result of their chemical changes during maturation. During wine oxidation, an increase in the concentration of eugenol, vitispirane (a compound belonging to terpenoid), and trans-1,8-terpin was observed [1, 13, 38]. A high concentration of eugenol may cause the aroma of wood, while vitispirane—the smell of camphor [1].

The concentration of volatile phenolic compounds changes during the wine oxidation; for example, 4-vinylguaiacol is converted to 4-(1-ethoxyethyl)phenol, while 4-ethylphenol oxidation results in the formation of numerous oligomerization, functionalization, and fragmentation products [60]. These compounds are important aromatizing compounds of wine [38].

Condensation of glycerol and acetaldehyde in an acidic environment results in acetal formation. This condensation leads to the formation of *cis*- and *trans*-5-hydroxy-2-methyl-1,3-dioxane as well as *cis*- and *trans*-4-hydroxymethyl-2-methyl-1,3-dioxalane. The smell of these substances is sensed as sweet and is characteristic for Port wine [13].

The oxidation of indole-3-acetic acid results in the formation of 2-formamidoacetophenone and 3-(2-formylaminophenyl)-3-oxopropionic acid, which can form 2-aminoacetophenone of a soap-like aroma [1].

As a result of 18-month-long maturation in barrels, a decrease in the concentration of ethyl butyrate and ethyl acetate was observed, while ethyl hexanoate and ethyl decanoate content was increased. As a result of the oxidation of Pinotage wines, the concentration of isoamyl acetate was decreased (creating banana notes which are characteristic for these wines) and development of a potato-like aroma was noticed [1].

The small amounts of oxygen which were inserted inside the bottles during the bottling and corking process increased the intensity of the fruit aromas of wines, which is probably related to the formation of compounds such as furaneol [61]. These also contribute to the lowering of SO_2_ concentration due to its reaction with the oxidation products of phenolic compounds and ethanol. The sulphur compounds whose level of drop is recorded in the presence of oxygen are DMS (dimethyl sulphide), hydrogen sulphide, and methyl mercaptan. The concentration of mercaptans falls most probably as a result of their oxidation to disulphides, which, however, has not been confirmed [13, 48]. Adequately low oxygen concentration allows the preservation of 3-mercaptohexanol and its acetate, compounds that create the aroma of Sauvignon Blanc wines. Under such conditions, the formation of sotolon and aldehydes is lower and the aroma is preserved, whereas the formation of small amounts of DMS may positively affect the aroma [62]. Nevertheless, through exposure to large amounts of oxygen after bottling, the concentration of compounds causing tropical fruit aromas (3-mercaptohexanol and 3-mercaptohexanol acetate) and floral aromas (e.g., linalool) decreases. The accumulation of compounds, such as aldehydes, lactones, and acetals, responsible for oxidation aroma also occurs, but the biggest effect on the bouquet is exerted by aldehydes and sotolon [62]. Higher concentrations of oxygen getting inside the bottle lead to the acceleration of the formation of 1,1,6-trimethyl-1,2-dihydronaphthalene, which is a compound characteristic of oxidized Riesling wines. As a result of these changes, wines lose their floral, citrus aromas and gain nutty and boiled vegetables notes [63].

Contact of wine with oxygen also has a significant impact on its colour. Oxygen in the case of white wine causes unfavourable browning, which is caused by three mechanisms of oxidation [10, 53, 64]:

1. It causes oxidation of phenolic compounds to the corresponding quinones, which are then polymerized, resulting in yellow-brown pigments. This reaction involves the iron and copper atoms that catalyze oxidation reactions. The main compounds associated with the browning of white wine are not only (+)-catechin, (-)-epicatechin, and dimers of proanthocyanidins B1-B4 but also coumarin and stearic, ferulic, and coffee acids, if they react with the mentioned compounds
2. It oxidizes tartaric acid to the glyoxylic acid forming bridges between the phenolic compounds, leading to their condensation, and copper and iron atoms are the catalysts. The colourless adducts of the two molecules of (+)-catechin connected through the carboxymethine bridge as a result of dehydration are transformed into yellow-brown xanthylium salts
3. It oxidizes phenolic compounds, resulting in hydrogen peroxide generation, which in turn oxidizes ethanol to acetaldehyde. Acetaldehyde forms bridges between (+)-catechin molecules (giving dimers joined by ethyl bridge) and also between dimers resulting from the oxidation of tartaric acid. This leads to the formation of brown pigments, the intensity of which is proportional to the degree of polymerization

Owing to the condensation reactions that are mediated by oxygen, the resulting polymerized dyes fall out in the form of a sediment and cause unacceptable changes in the appearance of the wine [39].

One of the disadvantages of white wine colour is also its pinking. It has been proposed that white wine pinking can be a result of the rapid conversion of accumulated flavenes to the corresponding red flavylium salts formed from the hydrolysis of leucoanthocyanins. The pink-coloured compounds resistant to pH changes and sulphur dioxide bleaching are formed when the level of free sulphur dioxide lowers, which leads to an increase of the relative amount of the anthocyanin red flavylium form and its polymerization. However, this colouration may be caused by other compounds and polymeric materials [65]. Pink pigments could be also probably derived from 2-S-glutathionyl-caftaric acid, formed as a result of the reaction of glutathione with o-quinones (produced through the enzymatic oxidation of the caffeoylacetic acid and p-coumaric acid). Further aging of such a wine in bottles causes its final browning [50].

The transformations of anthocyanins and tannins lead to colour changes of red wines: from reddish, characteristic of young red wine, to mauve or red-brown characteristic for mature wine [66]. According to [67], dye polymerization reactions do not depend on oxygen and are initiated due to elevated temperature. On the other hand, other studies [45, 51] showed the effect of oxygen on these phenomena by the following mechanisms:

1. Direct condensation reaction between tannins (nucleophilic C6 or C8 carbons of (+)-catechin, (-)-epicatechin, or procyanidins) and the electrophilic C4 carbon of anthocyanin. The colourless condensation products are oxidized to flavylium ion with an intense red colour [53, 54]
2. As a result of the addition reaction, in the acidic environment, the proton is added to the ethanal. The resulting electrophilic carbocation reacts with the C6 or C8 carbons of the flavanol and then, after dehydration, with the C8 carbon of anthocyanin (present in a colourless form). The compound formed as a result of these reactions may then be protonated to form coloured condensation products of anthocyanins and flavan-3-oles connected by an ethyl bridge. According to this mechanism, malvidin 3-glucoside reacts with various procyanidins [1]. Acetaldehyde can also mediate the condensation of anthocyanins leading to formation of oligomeric methylmethine-linked anthocyanins. These compounds are not stable and can undergo further reactions with malvidin 3-glucoside or carboxypyrano-malvidin-3-glucoside forming orange or blue pigments, respectively [9]
3. Because of the reaction between the vinylphenol molecule and the C4 and C5 carbons of anthocyanin, followed by the oxidation of the resulting compound, a pyran ring is formed. The reaction between the flavylium ion and the catechin molecule with a vinyl group at the C8 carbon produces pigments with a red-orange colour (pyranoantocyanins) and other compounds that are resistant to pH changes and decolourization by the SO_2_ [5]. The indirect products of polymerization reaction are ethylidene and vinyl-linked anthocyanins and flavanols and vinyl-pyranoanthocyanins [51]

The decreased concentration of monomeric anthocyanins observed during ageing results in a simultaneous increase in the concentration of polymerized compounds with a red colour due to the condensation of tannins and anthocyanins under the influence of oxygen [43]. The polymerized dyes have a higher colour intensity and are more resistant to pH changes, the influence of SO_2_, and the decomposition [45, 51].

Too high amount of oxygen combined with high temperature may in turn cause the decomposition of tannins and anthocyanins leading to a yellow colour development [5]. Glyoxylic acid together with 5-hydroxymethylfurfural may react directly with flavanols leading to the formation of yellow-orange xanthylium compounds [9].

Determining the amount of oxygen introduced that is appropriate for the proper maturation of wines to produce the right concentrations of volatile compounds, but without a negative colour and taste changes, is not easy, because it depends on many factors, such as pH and chemical composition of the wine [56]. The optimal moment of introducing the oxygen is also still under discussion [57]. The use of too high a dose may bring the opposite effect to the intended one (e.g., oxidation of phenolic compounds, increase of astringency, and formation of undesirable compounds), as well as favour the activity of harmful microorganisms which develop under aerobic conditions (e.g., acetic acid bacteria). The condensation of phenolic compounds due to oxidation can lead to [Parpinello et al., 2012]:

- the loss of colour
- formation of aldehydes due to oxidation and therefore altered aroma
- an increase in volatile acidity

## 5. Conclusions

Many factors influence the vinification process. Among them, more and more attention is paid to the role of oxygen, which indicates its importance in this process. Oxygen can affect the wine during many stages of its production, but the most significant effects can be observed during the early stages (harvest, pressing, and maceration) and maturation.

The role of oxygen during vinification is extremely complex. This is due to the fact that this element is used by both yeasts and bacteria and is a substrate for numerous chemical transformations of must, pulp, and maturing wine.

Oxygen plays an important role in the processes of yeast adaptation and synthesis of unsaturated fatty acids and sterols, which are key compounds that build cell membranes.

The processes of oxidation of must components are very complex and can be perceived as both positive and negative, which is most often associated with oxygen concentration. Low concentrations of oxygen can exert positive effects, while excess of oxygen can lead to too intense changes consequently adversely affecting the quality of wine. Oxygen can modify the sensory characteristics of wine. The influence of oxygen on the taste, aroma, and colour results mainly from its strong oxidizing properties. Phenolic compounds, terpenes, and other components of wine are susceptible to oxidation reactions, which consequently affect the wine composition.

Red wines contain higher concentrations of phenolic compounds; hence, these are easier to be oxidized, but on the other hand, their high level (despite oxidation) makes wines more resistant to oxygen. Therefore, the contact with oxygen in the case of red wines is not as undesirable as in the case of white wines. Oxygen in the production of white wines is generally perceived as an undesirable factor because it causes adverse changes in the aroma, colour, and taste of wine. Oxygen absorbed at various stages of white wine production may result in the formation of oxidized aroma (stale, vegetable, with a hint of dried apples, peel, and stale bread crust and sometimes pungent and chemical, with a flat, watery taste), and even small amounts affect the loss of characteristic fruity notes. The same amount for red wines, which have a significantly higher “oxygen tolerance,” is the minimum dose required to obtain the desired characteristics. Due to these differences, the impact of oxygen on wine is assessed differently depending on the type of wine.

New studies focus on the use of oxygen as a factor that allows obtaining the desired sensory characteristics; hyperoxygenation and microoxygenation consisting of purposeful contact with oxygen in low concentrations are more interesting. These techniques shorten the production process and affect the quality of products, which in turn brings economic benefits for winemakers.

## Figures and Tables

**Table 1:** The influence of oxygen on the initial stages of vinification [1, 4–7].

Adverse effects	Beneficial effects
Changes in taste, colour, and aroma of white wine due to degradation of phenolic compounds	Increased extraction of phenolic compounds from fruit tissues
Formation of deposit in red wine due to phenolic compound precipitation—reduction in the amount of phenolic compounds in the wine	Obtaining more balanced sensory characteristics of wine (such as lower acidity and astringency and increased colour intensity)
	Reduction of the amount of reducing aromas in wine (smell of mould, fungi, dirty cloth, rotten eggs, garlic, or cauliflower)

Hyperoxidation (9 mg O_2_/L)	
Lower intensity of aroma due to the formation of acetates and higher aldehydes (containing from five to ten carbons)	Better resistance of white wine to browning because of removal of proanthocyanidins
	Formation of higher concentrations of compounds favourably affecting the aroma of wine such as (i) six-carbon compounds (hexane-1-ol, 2-hexenal) (ii) higher alcohols (2-phenylethanol) (iii) fatty acids and their acetate and ethyl esters (hexyl, isoamyl, and 2-phenylethyl acetate and butyrate, decanoate, and ethyl hexanoate) (iv) volatile terpenes

**Table 2:** The influence of oxygen during the fermentation stage [1, 5, 24, 26, 29].

No oxygen	Oxygen addition in amount 5-20 mg O_2_/L
Decrease in fatty acid desaturase activity causes long-chain unsaturated fatty acids to not form	Appropriate lipid synthesis results in high glycolysis index and the highest possible yield of ethanol production

Inhibition of fermentation due to disorders in the synthesis of lipids	Appropriate synthesis of fatty acids and ergosterol implicates better yeast cell resistance to ethanol synthesized during fermentation and shorter fermentation time
	Reduction of the level of reducing aromas
	Formation of volatile compounds, such as esters (acetates and ethyl esters), higher alcohols, medium-chain fatty acids, branched acids, aldehydes, and ketones, whose presence at a concentration < 300 mg/L positively affects the complexity of wine aromas
	Faster conversion of malic acid into lactic acid

**Table 3:** Technological doses of oxygen present during wine maturation.

Technological process	Oxygen amount
Filtration	2-4 mg/L [10] 4-7 mg/L [1]
Centrifugation	0.95 mg/L [10] 8 mg/L [1]
Maturation in wood barrels	20-50 *μ*g/L [10]
Total Oxygen Package (TPO)	1-9 mg/L [[13]; [48]]
Aging in bottles	0.005-5 mg/L/year [48]
